# Pathophysiology of focal segmental glomerulosclerosis

**DOI:** 10.1007/s00467-006-0357-2

**Published:** 2007-03-01

**Authors:** Kimberly Reidy, Frederick J. Kaskel

**Affiliations:** 1 Division of Pediatric Nephrology, Children’s Hospital at Montefiore, Bronx, NY 10467 USA; 2 Division of Pediatric Nephrology, Children’s Hospital at Montefiore, 111 East 210th St., Bronx, NY 10467 USA

**Keywords:** Focal segmental glomerulosclerosis (FSGS), Nephrotic syndrome, Pathophysiology, Podocyte, Injury, Animal models, Nephrin, Podocin, Transforming growth factor (TGFβ), Tubulointerstitial

## Abstract

Focal segmental glomerulosclerosis (FSGS) is a major cause of idiopathic steroid-resistant nephrotic syndrome (SRNS) and end-stage kidney disease (ESKD). In recent years, animal models and studies of familial forms of nephrotic syndrome helped elucidate some mechanisms of podocyte injury and disease progression in FSGS. This article reviews some of the experimental and clinical data on the pathophysiology of FSGS.

## Learning objectives

- Discuss the experimental and clinical data on the pathophysiology of FSGS
- Review the alterations in glomerular structure and function associated with FSGS
- To identify potential mechanisms responsible for disease progression in FSGS
- Distinguish some targets for the future therapy of FSGS

Focal segmental glomerulosclerosis (FSGS) is a disease entity defined by findings on kidney biopsy [1, 2]. FSGS is the major cause of idiopathic steroid-resistant nephrotic syndrome (SRNS) in children and adults [3]. FSGS is the most common cause of acquired chronic renal insufficiency in children and frequently leads to progression to end-stage kidney disease (ESKD) [2]. FSGS may occur secondary to such disparate disease processes as HIV and obesity [1, 4]; this review focuses on the pathophysiology of primary FSGS (i.e., with no underlying illness).

**Fig. 1:**
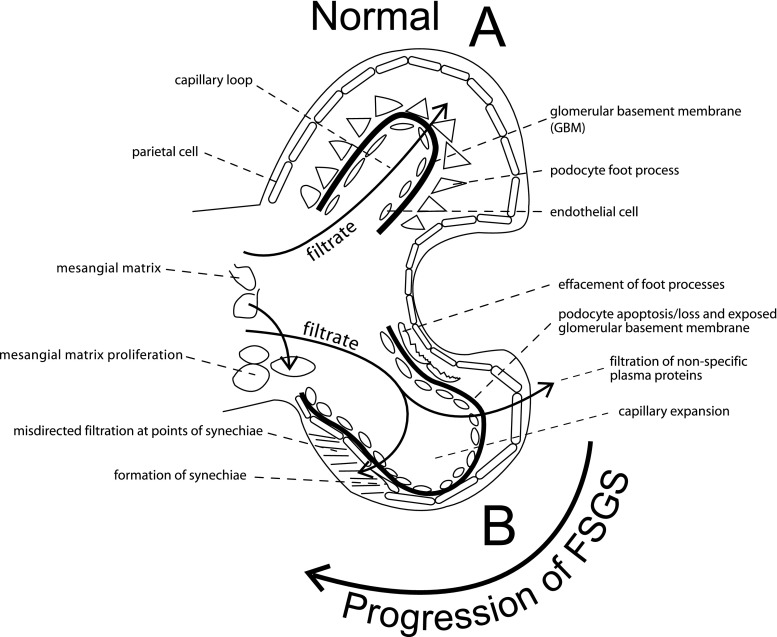
**A** Components of the normal glomerular filtration barrier: (1) glomerular basement membrane (GBM); (2) podocyte foot process; (3) endothelial cell; **B** Progressive changes seen in focal segmental glomerulosclerosis (FSGS) leading to sclerosis: (1) foot process effacement; (2) podocyte apoptosis/loss and exposed glomerular basement membrane; (3) filtration of non-specific plasma proteins; (4) capillary expansion; (5) formation of synechiae; (6) misdirected filtration at points of synechiae; (7) mesangial matrix proliferation. Adapted from Kwoh et al. [9]

Alterations of normal glomerular structure and function have been found in FSGS [5]. Normal function requires that the three major components of the glomerular filter (the endothelial cells, podocytes, and glomerular basement membrane) are intact and are able to provide a permselective filtration barrier (Fig. 1). Specialized tight junctions between podocyte foot processes create the slit diaphragm which is integral to preventing the loss of protein into the urinary space [6]. While the clinical presentation of FSGS is often heterogeneous, a characteristic feature of the disease is proteinuria, which implies the loss of this barrier [2, 7]. Indeed, electron microscopy has shown distortion of the normal architecture (or effacement) of the foot processes of podocytes in FSGS [1]. The connection between these projections of the epithelial cell and the underlying basement membrane can be disrupted, leading to the loss of nonspecific plasma proteins into the tubular filtrate [6].

However, unlike other causes of proteinuria and nephrotic syndrome, such as minimal change disease (MCD), FSGS often progresses to ESKD. While foot process effacement is seen in MCD as well as FSGS, histologically, FSGS is characterized by increased extracellular matrix within the glomerular tuft with obliteration of the glomerular capillary lumen. These sclerotic lesions occur focally and in only some segments of glomeruli, and are typically not associated with immune complex deposition [1]. The location of sclerotic lesions by light microscopy defines the variants of FSGS: perihilar variant (with sclerosis of the vascular pole), cellular variant (associated with hypercellularity of the capillary space), tip variant (involving the part of the glomerulus near the origin of the proximal tubule), and collapsing variant (with one or more glomeruli with global or segmental collapse) [1]. Clinically, the variants of FSGS differ; for example, the collapsing variant tends to progress more rapidly to ESKD and commonly occurs in the setting of HIV [1]. It is possible that different mechanisms may play a role in the pathogenesis of each variant of FSGS [7, 8].

Insight into the pathogenesis of FSGS developed over the past decade from studies of genetic mutations in mice, models of progressive glomerulosclerosis (such as the rat remnant kidney model), and identification of gene mutations in some familial forms of nephrotic syndrome (including congenital nephrotic syndrome and familial and autosomal dominant FSGS) [7, 9, 10].

Key in the pathogenesis of FSGS is podocyte damage and loss [5, 6]. Injury to podocytes occurs by four major mechanisms: alteration of the components of the slit diaphragm or interference with its structure, dysregulation of the actin cytoskeleton, alteration of the glomerular basement membrane or its interactions with the podocyte, or alteration of the negative surface charge of the podocyte [6, 9]. Damage to podocytes triggers apoptosis and their detachment of podocytes from the glomerular basement membrane [6, 9]. The ensuing reduction in podocyte number is felt to play an important role in the pathogenesis of FSGS [7]. The podocyte is normally a terminally differentiated cell with limited proliferative capacity in response to injury [7]. The initial insult to the podocyte leads to further damage mediated by cytokine release, mechanical stress, and further loss of polarity, resulting in sclerosis and scarring of the glomerulus [7, 9].

Genetic mutations seen in congenital forms of nephrotic syndrome and FSGS enabled researchers to identify specific gene mutations involved in podocyte damage [10]. Mutations of the nephrin gene, a podocyte-specific transmembrane component of the slit diaphragm, are found in congenital Finnish-type nephrotic syndrome, and may lead to loss of normal caliber slit diaphragms [6, 9–11]. In mouse models, mutations of nephrin-like transmembrane genes (*NEPH-1*) which also localize to the slit diaphragm result in proteinuria and early death [6, 10].

It is unclear how alteration of the slit diaphragm results in podocyte loss. The slit diaphragm may be integral to maintaining cell polarity or its damage may alter the balance of cell signals, resulting in apoptosis. Mutations in a Fyn kinase (one of the src tyrosine kinases) that phosphorylates nephrin and may regulate cell cycle and apoptosis resulted in proteinuria and foot process effacement in a mouse model [9, 10].

Other proteins which are part of the slit diaphragm complex include: podocin, CD2-associated protein (CD2AP), FAT, ZO-1, P-cadherin, an LAP (leucine rich repeat and PDZ domain) protein, and MAGI-1 [6, 10]. Mutations in podocin (a transmembrane protein that interacts with nephrin, NEPH-1 and CD2AP) have been identified in familial FSGS [9, 10, 12]. Recently, mutations in CD2AP, an immunoglobulin-like protein that is involved in nephrin integration with the podocyte cytoskeleton, have also been linked to genetic forms of FSGS [10, 13, 14]. In mouse models, the loss of FAT1 and FAT2 (transmembrane proteins with cadherin-like repeats) results in the absence of slit diaphragms, proteinuria, and early death [10]. The role of the other components of the slit diaphragm in the pathophysiology of FSGS is not yet clear.

Alpha-actinin-4, an important structural component of the podocyte cytoskeleton, is mutated in some autosomal dominant forms of FSGS [10, 15–17]. Other mutations have been identified in association with FSGS in addition to abnormal structural proteins. For example, TRPC6 is a cation-selective ion-channel protein that mediates calcium signals and has also been associated with FSGS [18].

Certain clinical variants of FSGS are suggestive of different mechanisms of injury to the podocyte. For example, a circulating factor which leads to glomerular basement membrane injury has been proposed in the pathogenesis of some types of FSGS [19, 20]. For example, there appears to be a role of a circulatory factor in the recurrence of FSGS in transplanted kidneys [20]. In some patients with recurrent FSGS, proteinuria remits in response to plasmapheresis and the removal of serum proteins. In addition, injections of serum from patients with recurrent FSGS were capable of inducing proteinuria in rats [20].

Another example of alternative mechanisms of injury is collapsing FSGS, which occurs in the setting of viruses such as HIV. In collapsing FSGS, dysregulation of the podocyte cell cycle appears to result in immature, proliferative podocytes [21, 22]. Finally, recent work has focused on the role of the parietal epithelial cell in the pathophysiology of FSGS [23]. Proliferation of parietal epithelial cells was identified in both a transgenic model of FSGS and a biopsy from a patient with collapsing FSGS [23].

Of great clinical importance is the mechanism by which the initial podocyte injury progresses to the final sclerotic lesion (Fig. 1). As podocyte numbers decline, there is a relative exposure of the glomerular basement membrane. Maladaptive interactions develop between the glomerular basement membrane and the parietal epithelial cells. Expansion of synechiae and/or the leak of protein into Bowman’s space results in the deposition of collagen. Ultimately, this results in the collapse of the capillary loop and the loss of endothelial cells [5].

**Fig. 2:**
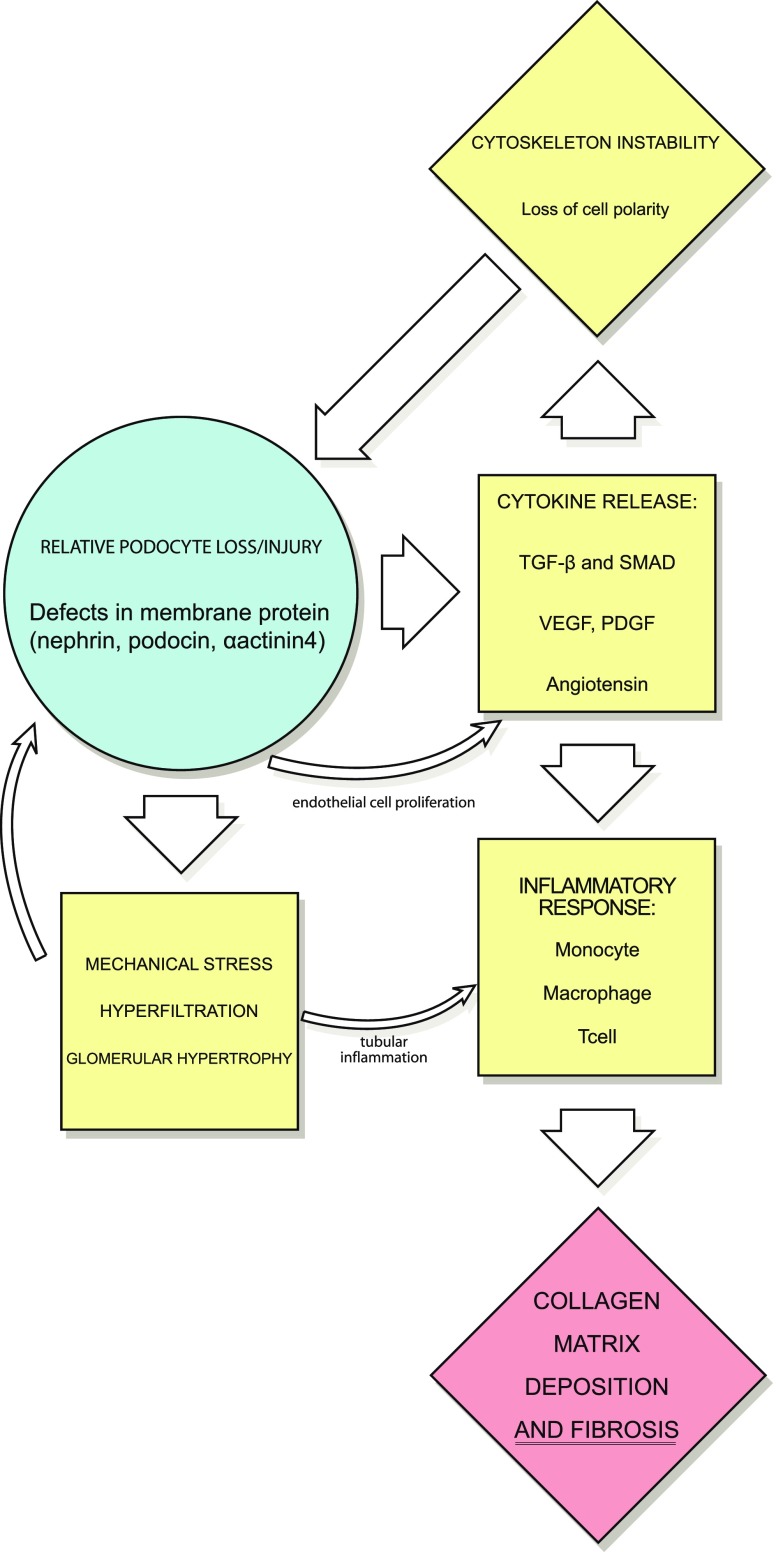
Factors involved in the progression of FSGS to end-stage kidney disease (ESKD): initial loss or injury to podocytes (related to defects in membrane proteins or cytoskeleton instability) leads to cytokine release, mechanical stress, hyperfiltration, and glomerular hypertrophy. These factors lead to upregulation of an inflammatory response mediated by monocytes, macrophages, and T-cells. The end result is collagen matrix deposition and fibrosis, and progression to ESKD

Factors resulting in the progression of FSGS to ESKD have also been the focus of recent research (Fig. 2). Cytokines and vasoactive factors are believed to play a major role in the progression of FSGS. The overexpression of transforming growth factor β (TGFβ) or its effector proteins, the Smads, leads to glomerulosclerosis in animal models [8, 24]. Activation of the renin-angiotensin system upregulates TGFβ and is felt to further lead to the progression of disease [7, 24]. Other angiogenic factors, such as platelet-derived growth factor (PDGF) and vascular endothelial growth factor (VEGF) may also play a role in disease progression [24]. The evidence for this is primarily based on animal models of progressive glomerulosclerosis, such as the rat remnant kidney model. In this model, PDGF and VEGF are upregulated and the later loss of VEGF expression correlates with progression of the glomerulosclerosis [24, 25].

Mechanical stress is also believed to play a role in the progression of FSGS [9, 26]. Increased filtration due the defects of the filtration barrier results in increased single-nephron glomerular filtration rate (SNGFR). This hyperfiltration results in hypertrophy of glomeruli. The hypertrophy exacerbates the mismatch between the glomerular basement membrane and the decreased numbers of podocytes, resulting in further injury [9].

Another factor in the progression of FSGS is tubulointerstitial injury. Clinically, tubulointerstitial injury is a predictor of the loss of renal function in FSGS [1, 27]. The nonspecific entry of proteins into the tubular lumen is one potential source of damage to the interstitium. Indeed, persistence of nephrotic-range proteinuria is a negative prognostic factor for the progression of FSGS to ESKD [28]. While it is unclear if proteinuria itself is toxic to the tubulointerstitium, decreases in proteinuria achieved by angiotensin-converting enzyme (ACE) inhibitors and by angiotensin receptor blockers (ARB) appear to slow disease progression in some adults with FSGS [9, 29].

The presence of plasma proteins in the tubular filtrate may directly injure the tubulointerstitium. Cytokines (such as TGFβ), when present in the tubules, will recruit monocytes, macrophage, and T-cells. This stimulates other cytokines, including interleukin-1, tumor necrosis factor alpha, and other chemokines [24]. The inflammatory infiltrate leads to mesangial matrix deposition, promoting the collapse of glomeruli. The cellular infiltrate and cytokines also damage tubular epithelial cells, and some tubular epithelial cells may undergo transformation to mesenchymal cells (an epithelial-mesenchymal transition or EMT) [24]. These mesenchymal cells, as well as recruited and stimulated fibroblasts, result in collagen matrix deposition and tubulointerstitial fibrosis [24].

The beneficial effects of blocking the renin-angiotensin system may not be limited to their antiproteinuric or antihypertensive effects. As noted earlier, angiotensin stimulates TGFβ, contributing to fibrosis. It can also induce oxidative stress and it is stimulated by mechanical stress, such as hyperfiltration [24]. In addition, angiotensin affects intracellular calcium concentrations and the podocyte cytoskeleton [24]. Inhibition of angiotensin may slow progression by these local mechanisms [9, 29].

With the increasing incidence of FSGS in children [30], these pathways of podocyte injury and disease progression provide important targets for future intervention. Trials have already been initiated to antagonize cytokines, such as TGFβ. Future therapeutic targets may include factors involved in podocyte protection or tubulointerstitial injury.

## Questions

(Answers appear following the reference list)

1. Which of the following statements is TRUE regarding the current understanding of the pathogenesis of focal segmental glomerulosclerosis (FSGS)?
  - FSGS may result from immune-complex-mediated damage to endothelial cells
  - Alterations in components of the slit diaphragm may play a role in the pathogenesis of FSGS
  - Proliferation of podocytes leads to cytokine release and mechanical stress, resulting in scarring and sclerosis of the glomeruli
  - Mutations in a chloride channel have been associated with FSGS and may be pathogenic
2. All of the following are mutations of structural proteins that have been identified as pathogenic in FSGS EXCEPT:
  - Sodium channel mutation
  - Alpha-actinin-4
  - Nephrin
  - Podocin
3. Progression of FSGS to end-stage kidney disease (ESKD) results from:
  - Downregulation of transforming growth factor β (TGFβ)
  - Decreased glomerular filtration
  - Tubulointerstitial injury
  - Blockade of the renin-angiotensin system
4. Proteinuria in the setting of FSGS:
  - Has no effect on clinical course
  - May be decreased by treatment with angiotensin-converting enzyme (ACE) inhibitors
  - Results from an increased number of glomerular foot processes
  - Leads to the loss of mesangial matrix
5. Which of the following is FALSE:
  - A circulating factor may play a role in the pathogenesis of FSGS
  - Proliferation of parietal epithelial cells has been identified in collapsing FSGS
  - Podocyte loss due to necrosis appears to play a role in the pathogenesis of FSGS
  - CD2-associated protein, FAT, nephrin, and podocin are examples of slit diaphragm proteins
